# Anthocyanins as Adjunctive Dietary Modulators of the Gut–Eye Axis: Bioavailability, Biotransformation, and Implications for Ocular Health

**DOI:** 10.3390/foods15132270

**Published:** 2026-06-24

**Authors:** Nicoleta Corina Predescu, Camelia Papuc, Georgeta Stefan, Petronela Mihaela Rosu, Mihail Chervenkov, Mihaela Saracila, Tatiana Dumitra Panaite, Iuliana Ionascu

**Affiliations:** 1 Faculty of Veterinary Medicine of Bucharest, University of Agronomic Sciences and Veterinary Medicine of Bucharest, 105 Splaiul Independentei, District 5, 050097 Bucharest, Romania; georgeta.stefan@fmvb.usamv.ro (G.S.); petronela.rosu@fmvb.usamv.ro (P.M.R.); iuliana.ionascu@usamv.ro (I.I.); 2 Academy of Romanian Scientists (AOSR), 54 Splaiul Independentei, District 5, 050094 Bucharest, Romania; cami_papuc@yahoo.com; 3 Faculty of Veterinary Medicine, University of Forestry, 10 Kliment Ohridski Blvd., 1700 Sofia, Bulgaria; vdmchervenkov@abv.bg; 4 Department of Plant and Fungal Diversity and Resources, Institute of Biodiversity and Ecosystem Research, Bulgarian Academy of Sciences, 1113 Sofia, Bulgaria; 5 National Research and Development Institute for Biology and Animal Nutrition, 077015 Balotesti, Romania; mihaela.saracila@ibna.ro (M.S.);

**Keywords:** anthocyanin metabolites, bioavailability, biotransformation, gut microbiota, functional berry, retinal pigment epithelium, gut–eye axis

## Abstract

Retinal diseases such as age-related macular degeneration (AMD) and diabetic retinopathy (DR) are major causes of visual impairment and are closely associated with oxidative stress, inflammation, vascular dysfunction, and metabolic imbalance. Increasing evidence suggests that gut microbiota also contributes to retinal homeostasis, supporting the emerging concept of the gut–eye axis. In this context, dietary anthocyanins—with blueberry anthocyanins serving as a primary representative model—have attracted attention as potential adjunctive nutritional modulators of ocular health. However, their biological effects are strongly influenced by their limited bioavailability and extensive gastrointestinal metabolism. The objective of this review is to analyze the gastrointestinal fate of dietary anthocyanins and to discuss how their absorption, enzymatic transformation, and microbial biotransformation may influence ocular protection through the gut–eye axis. The review summarizes current knowledge regarding anthocyanin stability in the oral cavity, stomach, small intestine, and colon, as well as the formation of circulating phenolic metabolites generated by the host and through microbial metabolism. In addition, the molecular mechanisms through which anthocyanins and their metabolites may support retinal health are examined, including antioxidant, anti-inflammatory, vasoprotective, and neuroprotective actions. Overall, dietary anthocyanins, illustrated through the rich profile of blueberries, represent promising adjunctive compounds for supporting ocular health, although further clinical and mechanistic studies are still required.

## 1. Introduction

Visual impairment and retinal diseases represent a major global public health challenge, affecting hundreds of millions of individuals worldwide and substantially reducing quality of life [1]. Among the most prevalent causes of vision loss are age-related macular degeneration (AMD), diabetic retinopathy (DR), glaucoma, and progressive myopia [2]. Although these disorders differ in their etiology and clinical manifestations, they share several common pathogenic mechanisms, including oxidative stress, chronic inflammation, mitochondrial dysfunction, vascular impairment, and neurodegeneration [2]. Current therapeutic approaches, such as anti-vascular endothelial growth factor (anti-VEGF) therapy, laser photocoagulation, and corticosteroid administration, have significantly improved clinical outcomes in selected patients [3]. Nevertheless, these interventions do not fully prevent disease progression and are often associated with high costs, invasive administration, or variable therapeutic responses [3]. Consequently, increasing attention has been directed toward nutritional and lifestyle-based strategies that may complement conventional therapies and support long-term ocular health [4].

Recent advances in microbiome research have led to the emergence of the gut–eye axis, a concept describing the bidirectional interactions between intestinal homeostasis and ocular physiology [5,6,7]. Some evidence suggests that alterations in gut microbial composition and function may contribute to retinal disorders through mechanisms involving immune dysregulation, impaired intestinal barrier integrity, systemic inflammation, altered production of microbial metabolites, and oxidative stress [5,6]. Also, dietary interventions capable of modulating gut microbiota may influence retinal health and visual function [7]. Understanding the molecular pathways linking the gastrointestinal tract and the eye has therefore become an important area of investigation in ophthalmology and nutritional science [5,6,7].

Among dietary bioactive compounds, anthocyanins have attracted considerable interest because of their potent antioxidant, anti-inflammatory, vasoprotective, and neuroprotective properties [8,9]. Anthocyanins are water-soluble flavonoid pigments responsible for the red, blue, and purple coloration of many fruits, vegetables, and cereals [8]. Experimental and clinical studies have demonstrated that anthocyanins used as adjuvants can modulate multiple biological pathways associated with retinal protection, including reactive oxygen species scavenging, regulation of inflammatory signaling cascades, improvement of endothelial function, modulation of angiogenesis, and enhancement of endogenous antioxidant defenses [10,11,12]. In association, anthocyanins may influence glucose metabolism and gut microbial composition, suggesting that some of their ocular benefits may be mediated indirectly through the gut–eye axis [13,14].

Although anthocyanins are widely distributed among berries, grapes, cherries, blackcurrants, purple corn, black rice, and other pigmented plant foods, blueberries represent one of the richest and most extensively investigated dietary sources [15]. For this reason, blueberries serve as an ideal model matrix to investigate the systemic behavior of dietary anthocyanins. They contain a particularly diverse anthocyanin profile, comprising glycosides of malvidin, delphinidin, cyanidin, petunidin, and peonidin [16]. Furthermore, blueberry anthocyanins have been repeatedly associated with improvements in vascular function, cognitive performance, metabolic health, and visual function [11]. The diversity of their anthocyanin composition and the growing body of experimental and clinical evidence make blueberries an invaluable paradigm for investigating the broader relationships between general anthocyanin metabolism and ocular health [17,18].

Despite their promising biological activities, the health effects of anthocyanins are strongly influenced by their limited bioavailability and extensive metabolism [19]. Following ingestion, anthocyanins undergo a complex series of transformations involving chemical degradation, enzymatic modification, intestinal absorption, hepatic conjugation, enterohepatic circulation, and microbial biotransformation within the colon [20]. These processes generate a broad spectrum of metabolites, many of which may contribute substantially to the biological effects traditionally attributed to the parent compounds [20]. So, understanding the gastrointestinal fate of anthocyanins is essential for elucidating the mechanisms through which they may influence retinal physiology and pathology [21].

The present review examines the potential role of dietary anthocyanins as candidate nutritional modulators of the gut–eye axis, utilizing blueberries as a primary illustrative model. Particular emphasis is placed on their gastrointestinal bioavailability, enzymatic and microbial biotransformation from the oral cavity to the colon, the generation of bioactive metabolites, and the molecular mechanisms through which these compounds contribute to ocular protection. In addition, current evidence regarding the effects of anthocyanins and their metabolites on retinal structure, retinal function, and major ocular disorders is critically discussed, highlighting both current knowledge and future research directions.

A comprehensive literature search was conducted across the PubMed, Web of Science, Scopus, and Google Scholar databases to identify relevant peer-reviewed articles published up to May 2026. The search strategy utilized combinations of the following keywords: ‘anthocyanins’, ‘blueberry anthocyanins’, ‘gut–eye axis’, ‘microbiota biotransformation’, ‘retinal protection’, ‘bioavailability’, and ‘phenolic metabolites’. Only articles written in English were considered. The selection process prioritized randomized controlled clinical trials, in vivo animal models, and mechanistic in vitro studies that directly evaluated the metabolic fate of anthocyanins and their functional implications on ocular tissues. Meta-analyses and highly relevant previous reviews were also screened for cross-references.

## 2. Ocular Disorders and the Need for Novel Nutritional Strategies

Vision-threatening ocular disorders represent a major global public health challenge, affecting hundreds of millions of individuals worldwide [1]. The retina is characterized by high oxygen consumption, intense metabolic activity, and continuous exposure to light [22]. Increasing evidence indicates that oxidative stress, chronic inflammation, mitochondrial dysfunction, and vascular impairment are central mechanisms involved in the pathogenesis of numerous retinal diseases, including age-related macular degeneration (AMD), diabetic retinopathy (DR), retinal ischemia, and light-induced retinal injury [17,18,23].

### 2.1. Age-Related Macular Degeneration (AMD)

Age-related macular degeneration (AMD) is a common progressive eye disease that damages the macula—the central part of the retina responsible for sharp, straight-ahead vision required for reading, facial recognition, and detailed visual tasks—and is the leading cause of irreversible vision loss among elderly individuals in developed countries [2,17]. The global prevalence of AMD is expected to increase substantially due to population aging, with projections suggesting that nearly 300 million people may be affected by 2040 [24].

AMD is generally classified into two forms: dry (atrophic) AMD and wet (neovascular) AMD [25]. Dry AMD accounts for approximately 85–90% of all cases and is characterized by the gradual accumulation of extracellular deposits known as drusen beneath the retinal pigment epithelium (RPE), followed by progressive degeneration of photoreceptors and RPE cells [26]. Wet AMD, although less common, is responsible for most cases of severe vision loss and is characterized by choroidal neovascularization, leakage, hemorrhage, and rapid retinal damage [25].

Oxidative stress is considered one of the principal pathogenic factors in AMD development [27]. The retina is highly susceptible to oxidative damage because of its elevated oxygen consumption and constant exposure to visible light [28]. Reactive oxygen species (ROS) generated through retinal mitochondrial metabolism, as well as photo-oxidative reactions, overwhelm endogenous antioxidant defenses [23]. Retinal pigment epithelial cells are particularly vulnerable to oxidative injury, which accelerates cellular senescence and apoptosis [29].

Chronic inflammation further contributes to AMD progression [30]. Oxidative damage stimulates activation of the complement system and innate immune responses within the retina [30]. Activated microglia and infiltrating macrophages release pro-inflammatory cytokines, including tumor necrosis factor-alpha (TNF-α), interleukin-1β (IL-1β), and interleukin-6 (IL-6), creating a sustained retinal inflammatory microenvironment [30]. Retinal degeneration represents the final consequence of these interconnected pathological processes.

### 2.2. Diabetic Retinopathy

Diabetic retinopathy (DR) is one of the most common microvascular complications of diabetes mellitus and remains a leading cause of blindness among working-age adults worldwide [13]. Persistent hyperglycemia is the primary initiating factor in DR pathogenesis. Elevated glucose levels activate multiple biochemical pathways, including the polyol pathway, protein kinase C signaling, advanced glycation end-product (AGE) formation, and hexosamine pathway activation [12]. These metabolic disturbances increase oxidative stress and promote chronic retinal inflammation [5].

Vascular dysfunction is a hallmark of diabetic retinopathy [31]. Hyperglycemia-induced oxidative stress damages retinal endothelial cells and pericytes, leading to breakdown of the blood–retina barrier, increased vascular permeability, and capillary degeneration [5,31]. As a result, retinal edema, microaneurysm formation, and ischemic changes develop progressively. Impaired retinal blood flow further exacerbates tissue hypoxia and oxidative injury [10].

Vascular endothelial growth factor (VEGF) plays a critical role in DR progression [14]. Retinal hypoxia stimulates VEGF production by retinal cells, promoting pathological angiogenesis and increased vascular permeability [32]. Excessive VEGF expression contributes directly to diabetic macular edema and proliferative diabetic retinopathy, the most severe form of the disease [32]. Newly formed blood vessels are fragile and prone to leakage and hemorrhage, often resulting in substantial vision loss [14,32].

### 2.3. Other Retinal Disorders Associated with Oxidative Stress

Myopia, both high or pathological, has been associated with increased oxidative stress within retinal and scleral tissues [33,34]. Excessive axial elongation of the eye induces structural changes that may compromise retinal integrity and increase susceptibility to oxidative damage [35]. Experimental studies suggest that elevated ROS production contributes to retinal remodeling and photoreceptor dysfunction in myopic eyes [35].

Retinal ischemia occurs when retinal blood supply is reduced or interrupted, leading to oxygen deprivation and metabolic stress [36]. Restoration of blood flow often triggers ischemia–reperfusion injury, characterized by massive ROS generation, mitochondrial dysfunction, inflammatory activation, and neuronal apoptosis [36]. Retinal ganglion cells are especially vulnerable to ischemic injury, which may result in permanent vision loss [37].

Light-induced retinal injury represents another condition strongly associated with oxidative damage [38]. Prolonged exposure to intense visible or blue light increases ROS formation within photoreceptors and retinal pigment epithelial cells [28].

### 2.4. Current Therapeutic Approaches and Their Limitations

Current treatments for retinal diseases primarily target advanced pathological manifestations rather than the underlying mechanisms responsible for disease initiation and progression.

Despite significant advances in ophthalmological therapies, many retinal disorders remain progressive and multifactorial diseases in which oxidative stress, inflammation, vascular dysfunction, and metabolic imbalance continue to play central pathogenic roles [3,39,40]. Current therapeutic approaches have significantly improved clinical outcomes, but many treatments are invasive, expensive, and primarily target advanced stages of disease (Table 1). In these circumstances, growing attention has been directed toward nutritional strategies employing natural bioactive compounds capable of modulating oxidative stress and inflammatory pathways on the retina, thereby providing complementary or preventive therapeutic benefits [41].

Blueberries were selected as the primary focus of this review because they represent one of the richest dietary sources of anthocyanins and one of the most extensively investigated berry matrices in studies addressing visual function, retinal protection, gut microbiota modulation, and anthocyanin metabolism [21]. Therefore, blueberry anthocyanins provide a particularly suitable model for exploring the mechanistic links between gastrointestinal biotransformation, microbial metabolism, and ocular health [22].

## 3. Blueberry Anthocyanins as Candidate Nutritional Modulators of the Gut–Eye Axis

Anthocyanins still remain bioactive compounds with many unknown metabolic actions despite decades of intensive research [8]. They exhibit negligible systemic bioavailability in their intact form [9,42]; still, they are consistently associated with beneficial health outcomes [15,19]. This apparent contradiction has prompted a re-evaluation of the well-known frameworks used to assess their biological relevance. Early investigations emphasized the antioxidant capacity of anthocyanins, often interpreting their health effects through a radical-scavenging perspective [16]. Lately, researchers have shifted toward a broader and more integrative view, recognizing anthocyanins as regulators of host metabolism [43], modulators of gut microbial homeostasis [44], inflammation-related pathways [45], cellular signaling networks [10], and enhancers of vascular endothelial function [11].

**Figure 1:**
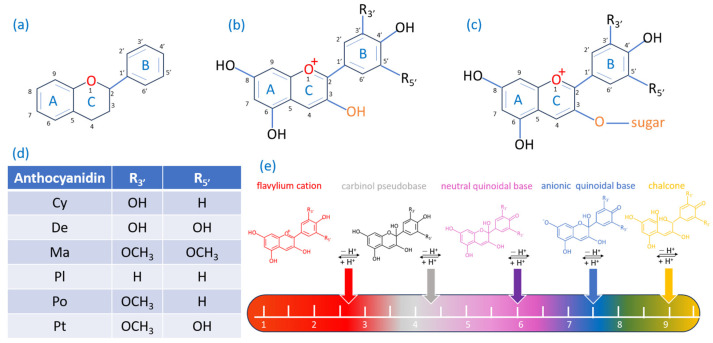
Anthocyanidin structures and pH-dependent transformations. Common skeleton of flavonoids (**a**). General structure of anthocyanidin (**b**). General structure of anthocyanin (**c**). Structure for common anthocyanidins (**d**). Anthocyanidin structure depending on pH (**e**).

Anthocyanins are glycosylated derivatives of anthocyanidins (aglycones) and share a common structural core known as the 2-phenyl-benzopyrylium chromophore with a characteristic C_6_–C_3_–C_6_ flavonoid skeleton (Figure 1a). The most common aglycone anthocyanidins found in blueberries are cyanidin (Cy), delphinidin (De), malvidin (Ma), pelargonidin (Pl), peonidin (Po), and petunidin (Pt) (Figure 1d). Their common structure comprises two benzene rings (A and B) linked through a pyran ring (C). The extended π-conjugation of this system, together with oxygen positive charge of the heterocycle and multiple hydroxyl and methoxy groups, enables anthocyanidins (Figure 1b) to absorb visible light and produce a wide range of colors. Also, the number and position of hydroxyl and methoxy substituents influence the structural diversity among anthocyanidins (Figure 1d). Sugar moieties—most commonly glucose, galactose, arabinose, and rhamnose—are typically attached at the C3 position of the anthocyanin (Figure 1c). The basic flavylium cation can be modified with different hydroxyl or methoxy groups at various positions, leading to the diverse structures. These substitutions, along with acylation of the sugar, strongly influence anthocyanins color stability, solubility, and biological behavior [46]. Anthocyanins are natural, water-soluble pigments that exist in an equilibrium of different structural forms, which are strongly influenced by the pH of the surrounding environment (Figure 1e). The color varies from red to purple, blue, and yellow, driven by electronic structural transformations involving the protonation (gain of H^+^) and deprotonation (loss of H^+^) of the anthocyanin molecule [47].

In the context of human nutrition, anthocyanins are abundant in blueberries, which are consumed as fresh treats or prepared as juice, puree, and jams or included in different cooked products [48,49]. According to some studies, malvidin glycosides and cyanidin glycosides are the most abundant in fresh blueberry and are present in higher concentrations in the blood and tissue after their ingestion; at the opposite site, the pelargonidin glycosides are absent or they are identified in trace amounts in the blood or other tissues [49,50]. This compositional profile may be relevant for their biological activity, as these compounds have long been associated with antioxidant-related benefits [15], including modulation against oxidative stress [11]. However, emerging evidence suggests that such effects alone cannot fully explain their physiological impact. For example, anthocyanin-rich foods such as blueberries have been linked to improved visual function [17], including enhanced retinal blood flow, protection of retinal cells, and potential reduction in the risk of age-related macular degeneration (AMD) [18]. Nevertheless, these outcomes are unlikely to result solely from the direct antioxidant activity of intact anthocyanins, given their limited absorption and rapid degradation in the upper gastrointestinal tract [51]. Consequently, the metabolic fate of anthocyanins has become a major focus of current research.

A defining feature of blueberry anthocyanins is their extensive biotransformation following ingestion along the digestive tract, starting in the oral cavity and ending in the colon. Although small amounts may be absorbed intact in the oral cavity, stomach, and small intestine, the majority of dietary anthocyanins are subjected to a hybrid enzymatic degradation [51]. Through this process, anthocyanins are converted into a diverse array of phenolic metabolites (specifically, phenolic acids, phenolic aldehydes, and other low-molecular-weight compounds). Importantly, these derived metabolites display greater chemical stability and prolonged systemic persistence compared with their parent compounds, converging towards a greater total bioavailability [9].

Although anthocyanins are rapidly absorbed after the oral administration of blueberry extract, their direct bioavailability in humans is extremely low, with only a small fraction of the initial compounds detected in plasma and urine [52]. Most circulating anthocyanins are present in their conjugated form, particularly malvidin and peonidin glucuronides. It is important to note that the metabolism of these compounds leads to the formation of numerous degradation products derived from anthocyanins, which appear in concentrations significantly higher than the original molecules [21]. These metabolites, detected at significantly higher levels in plasma and urine, considerably increase the estimated overall bioavailability when included in the calculation [52]. As evidenced through their detection in ileostomy fluid, only 30% of ingested anthocyanins reach the small intestine intact, where their concentrations remain relatively stable during intestinal transit [52]. The remaining fraction undergoes metabolic transformation, generating several degradation products such as gallic acid and protocatechuic acid in the intestinal environment, while compounds like syringic acid and vanillic acid predominate in systemic circulation [52]. Among these metabolites, phloroglucinol aldehyde exhibits the longest persistence in plasma. The comparison between healthy individuals and patients with ileostomy highlights the important role of an intact gastrointestinal tract in the metabolism and bioavailability of anthocyanins, suggesting that the intestine could represent a key site of action for these bioactive compounds [52].

Accumulating evidence indicates that these metabolites, rather than intact anthocyanins, account for a substantial proportion of the observed biological effects, including modulation of inflammatory responses, oxidative balance, lipid metabolism, and blood homeostasis [9]. Consequently, the interaction between anthocyanins and gut microbiota has emerged as a central determinant of their health-promoting properties. This bidirectional relationship not only governs the metabolic fate of anthocyanins but also influences microbial composition and functionality, ultimately shaping host physiological responses [20].

Taken together, these findings support a conceptual shift away from evaluating anthocyanins solely based on classical bioavailability metrics. Instead, their biological significance should be understood within an integrating system-level framework that includes food matrix effects, microbial metabolism, metabolite-mediated signaling, and host responses [53].

### Apparent and Total Bioavailability of Anthocyanins

Classical definitions of bioavailability emphasize the absorption of intact compounds and their measurable presence in systemic circulation [9,19,42]. Although appropriate for chemically stable molecules, this concept is less suitable for anthocyanins, which undergo extensive degradation and biotransformation during gastrointestinal transit [54]. Only a small proportion of ingested anthocyanins reaches the circulation in their native form, whereas a substantial fraction is converted into phase II conjugates and microbiota-derived phenolic metabolites. Increasing evidence suggests that these metabolites may contribute significantly to the biological effects attributed to anthocyanins, including antioxidant, anti-inflammatory, and vasoprotective activities relevant to ocular health. Therefore, assessment of anthocyanin bioavailability should extend beyond the measurement of intact compounds and include the generation and systemic actions of their metabolites [54]. As clearly defined by Ayvaz and his colleagues (2022) [21], anthocyanin bioavailability refers to the proportion of a bioactive compound that becomes available at a specific organ/tissue following absorption from the oral–gastrointestinal tract [21]. Regarding anthocyanins, the scientific publications distinguished two concepts of bioavailability: total bioavailability and apparent bioavailability [55]. Total bioavailability encompasses the fraction of a compound absorbed through the oral–gastrointestinal barrier that reaches the systemic circulation either in its native form or as metabolites generated during first-pass metabolism, whereas apparent bioavailability refers specifically to the fraction of anthocyanins absorbed intact [55]. Anthocyanin metabolism occurs along the entire gastrointestinal tract and includes enzymatic transformations within the oral cavity, stomach, and intestinal epithelial cells, as well as metabolic processes mediated by microorganisms residing in the oral cavity along to intestinal compartments [19]. Anthocyanins that escape absorption in the small intestine pass into the colon, where they encounter dense gut microbiota, leading to further, more extensive degradation, known as second-pass metabolism [21].

Bioaccessibility is the fraction of a nutrient or compound released from the food matrix during gastrointestinal digestion, making it available for absorption in the gut [56]. In this context, for blueberry anthocyanins as natural compounds, bioaccessibility is strongly influenced by food processing and the digestive environment. Comparative analyses of fresh, frozen–thawed, and smoothie preparations have shown that the disruption of the fruit matrix significantly enhance anthocyanins release during digestion [56]. Although blueberries contain a wide diversity of anthocyanins, less than 10% of the total content becomes bioaccessible during the oral phase. Simulated mastication resulted in significantly greater anthocyanin release than in vivo mastication, suggesting that in vitro oral digestion models may overestimate anthocyanins’ bioaccessibility and should therefore be interpreted with caution [56]. Frozen–thawed blueberries exhibited greater oral anthocyanin release than fresh fruits, while blending them into a smoothie resulted in the highest bioaccessibility, likely due to extensive structural disruption of the plant tissue. Gastric digestion further increased anthocyanin release for all preparations, reflecting their relative stability under acidic gastric conditions [56]. However, anthocyanin recovery declined markedly during the duodenal phase. This reduction is commonly attributed to the increased intestinal pH, which promotes structural instability and degradation of anthocyanins [56]. Nevertheless, the decrease in recovery may also reflect concurrent absorption processes occurring in the upper small intestine, where nutrients are efficiently absorbed. Therefore, the lower anthocyanin concentrations detected during the duodenal phase are likely the result of a combination of chemical instability, enzymatic transformation, and intestinal uptake rather than degradation alone [56]. Together, these concepts highlight the complexity of transforming anthocyanin intake into biological activity and underscore the importance of considering both bioavailability and bioaccessibility when evaluating their health effects [56].

According to a study from 2021, performed by Gonçalves and his colleagues, the estimated daily intake of anthocyanins in humans shows wide variability, ranging from only a few milligrams to several hundred milligrams per day [57]. In European populations, reported dietary anthocyanin intake varies considerably, from approximately 19.8 mg/day in men from the Netherlands to 64.9 mg/day in men from Italy, and from 18.4 mg/day in women from Spain to 44.1 mg/day in women from Italy. Outside Europe, average daily intakes are estimated at around 12.5 mg/day in the United States, 24.2 mg/day in Australia, and 37 mg/day in several Asian countries [57]. This variability reflects the inherent difficulty in accurately assessing anthocyanins consumption and is influenced by multiple factors, including dietary habits, gender, the presence of food intolerances, and the natural variability of anthocyanins content in foods. Intake levels are generally higher in populations that consume a Mediterranean diet, which is rich in red and blue-colored fruits, particularly berries, as well as red wine [57]. Although anthocyanins are classified as non-essential dietary compounds, their potential health-promoting properties have prompted efforts to define intake recommendations [58]. As an example, in China it was proposed that a daily intake of approximately 50 mg per person was effective, based on evidence suggesting a role for anthocyanins in reducing oxidative stress and, consequently, the risk of cancer, metabolic syndrome, diabetes, degenerative disorders, and other chronic diseases [58]. Although intact anthocyanins generally reach only low concentrations in systemic circulation, their consumption has consistently been associated with beneficial health outcomes. This observation suggests that the biological effects of anthocyanins may not depend exclusively on the parent compounds but could also involve bioactive metabolites generated through host metabolism and gut microbial biotransformation [57].

Dietary anthocyanins are ingested within a complex food matrix and are first subjected to oral and gastrointestinal digestion, during which a fraction of the compounds is released from the matrix, defining their bioaccessibility [56]. Bioaccessible anthocyanins may then be absorbed intact directly from the gastrointestinal tract or undergo extensive first-pass metabolism, including enzymatic transformations in the oral cavity, stomach, and intestinal epithelial cells, as well as biotransformation mediated by the gut microbiota [58]. As a result, anthocyanins reach systemic circulation either as intact molecules (apparent bioavailability) or as a diverse range of metabolites (total bioavailability). These circulating compounds and metabolites may subsequently exert biological effects at target tissues, contributing to the reported antioxidant, anti-inflammatory, cardiometabolic, and neuroprotective properties of anthocyanins [52].

**Figure 2:**
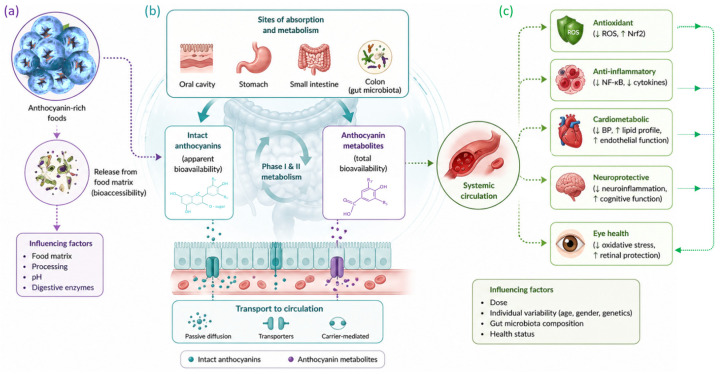
Schematic overview of anthocyanins’ bioaccessibility and systemic bioavailability in humans, including (**a**) dietary intake and gastrointestinal bioaccessibility, (**b**) absorption and metabolic transformation, and (**c**) systemic bioavailability and biological effects with emphasis on blueberry anthocyanins.

Current knowledge regarding anthocyanins’ absorption and transport in humans remains limited and challenging to assess, largely due to the rapid metabolism and structural diversity of these compounds [52]. Anthocyanins have long been regarded as having poor bioavailability. This lower apparent bioavailability ranges from less than 1% to approximately 2%, as only trace amounts of intact parent compounds are typically detected in the systemic circulation or in putative target tissues [54]. However, this apparent low bioavailability may be substantially underestimated when analytical approaches focus exclusively on native anthocyanins or a limited set of phenolic degradation products. Mouth–gastric–intestinal metabolism, occurring prior to absorption, generates a wide diversity of metabolites, including polar, water-soluble phase I and phase II conjugates and products derived from gut microbial activity [21]. When unmetabolized parent compounds and their diverse metabolic derivatives are collectively considered, the total bioavailability of anthocyanins appears to be considerably greater than previously assumed, as schematically illustrated in Figure 2, which summarizes the most important centers of the gastrointestinal tract where anthocyanins are absorbed intact or multiple metabolic pathways contribute to systemic exposure.

After dietary intake, anthocyanins are released from the food matrix—where they are bioaccessible—and absorbed along the gastrointestinal tract, where they exist as intact compounds or are transformed by host and gut microbiota. These compounds and their metabolites enter systemic circulation and exert multiple biological effects, modulated by individual and dietary factors (Figure 2).

Nonetheless, the relative contribution of each parent compound or metabolite class to the maintenance of human health and disease prevention remains an active area of investigation. In this context, the concept of metabolic availability—encompassing the generation, persistence, and biological activity of host- and microbiota-derived metabolites—offers a more relevant framework for interpreting anthocyanins’ functionality [50].

## 4. Intact Anthocyanins and Their Metabolites in a Diet–Microbiota–Host Context

### 4.1. Oral Processing and Initial Fate of Anthocyanins

Emerging evidence suggests that the oral cavity is not merely a passive transit compartment for anthocyanins but may represent an initial site of biochemical transformation [59]. Interactions with salivary enzymes, oral microbiota, and the oral mucosa can influence anthocyanin stability and promote the formation of early metabolites before the compounds reach the stomach. These processes may affect the subsequent bioavailability and metabolic fate of anthocyanins throughout the gastrointestinal tract [59]. Anthocyanins’ stability in the oral environment is strongly dependent on molecular structure. Therefore, delphinidin and petunidin glycosides exhibit greater susceptibility to degradation compared to cyanidin, peonidin, and malvidin glycosides during oral exposure [29]. The higher number of hydroxyl (HO-) groups on the B-ring of delphinidin makes it less stable, whereas methoxylation (addition of -OCH_3_ groups) on the B-ring—characteristic of malvidin, peonidin, and petunidin—increases stability. These differences highlight the influence of aglycone hydroxylation patterns and glycosylation on anthocyanins’ stability. Indeed, this sustains that anthocyanins’ compound-specific transformation begins in the oral cavity, before gastric or intestinal processing [59]. A research study detected anthocyanins in buccal mucus and epithelial cells shortly after ingestion. This study provides direct evidence of their interaction with oral tissues and suggests that local exposure and potential uptake occur at a very early stage [59]. The observed decline in anthocyanin levels during oral retention likely results from multiple concurrent mechanisms, including spontaneous chemical degradation, enzymatic metabolism by oral microbiota, adsorption to oral surfaces such as teeth and mucosal layers, and interactions with epithelial tissues [60]. Although some microbial metabolites and small bioactive compounds may gain systemic access through the oral mucosa, evidence demonstrating significant direct systemic absorption of anthocyanins or their metabolites from the oral cavity remains scarce [60]. Further studies are needed to clarify the contribution of this pathway to overall anthocyanin bioavailability. These processes position the oral cavity as a primary site of first-pass metabolic transformation [60]. Also, oral microbiota plays a functional and modulatory role in this process, as demonstrated by intervention studies showing that oral microbial activity can significantly influence anthocyanins’ stability and transformation dynamics. A crossover intervention study demonstrated a reduction of around 15% in anthocyanin loss, confirming that oral microbiota actively modulates anthocyanin fate [61]. Oral cavity serves as a first degradative force and may facilitate the formation of smaller bioactive metabolites through oral microorganisms, enhancing their biological availability to host tissues [59]. Research studies support the existence of an oral microbiota–anthocyanin interaction. This represents an early and determinant element of anthocyanins’ bioavailability. Furthermore, substantial variability in anthocyanins’ stability and metabolism has been observed, reflecting differences in oral microbial composition and activity [59]. This variability accentuates the importance of microbiome-dependent metabolism and supports a new approach to personalized bioavailability. Considering this, individual microbial profiles play a decisive role in shaping the metabolic fate and biological effects of dietary anthocyanins [61].

The available evidence clearly demonstrates that the oral cavity constitutes an active and functionally significant site for the early metabolism of anthocyanins, driven by both enzymatic and physicochemical processes. The rapid loss of intact anthocyanins in saliva is primarily mediated by enzymatic hydrolysis, particularly through the activity of β-glycosidases secreted by oral microbiota and epithelial cells [62]. This process is strongly influenced by anthocyanin molecular structure, as both aglycone composition and glycosylation patterns determine their susceptibility to degradation. Under the slightly acidic to neutral conditions of saliva, anthocyanins undergo structural rearrangements into hemiacetal and chalcone intermediates, facilitating cleavage of the heterocyclic C-ring [60]. The aglycones are characterized by increased lipophilicity and exhibit greater potential for local absorption but are unstable and rapidly degradable into smaller phenolic compounds, particularly protocatechuic acid (PCA) and phloroglucinol aldehyde (PGA) [59]. Immunohistochemical evidence confirms the presence of relevant metabolic enzymes and transporters within oral epithelial tissues, with anthocyanin-derived metabolites detectable within minutes of exposure [59]. These findings establish that anthocyanin metabolism begins immediately upon ingestion, through coordinated interactions between oral microbiota and host epithelial cells. Some of the enzymes involved in the oral cavity reactions are presented in Table 2. This early metabolic transformation generates bioactive phenolic metabolites that may contribute to systemic biological effects, highlighting the oral cavity as a previously underrecognized but critical determinant of anthocyanins bioavailability and functional activity [59]. The oral cavity degrades anthocyanins, driven by the presence of β-glucosidase, which is produced by both the oral microflora and the oral epithelial tissues. This enzyme breaks down the glycosidic bonds of anthocyanins, converting them into their corresponding aglycones. Some studies suggest that glycosylation, due to near neutral pH, produces colorless chalcone glucosides (via C-ring opening) from anthocyanins and further into phenolic acids, such as PCA, which are considered highly bioavailable and functional antioxidant agents [59,60]. Also, the oral degradation of anthocyanins is sustained by lactase phlorizin hydrolase (LPH). It is found in the epithelial cells of the oral mucosa and can act as both as β-galactosidase and as β-glucosidase (acting on phlorizin and similar flavanols). It specifically assists in the hydrolysis of flavonoid glycosides, including anthocyanins [63]. In the oral cavity, quinoid anhydrolase acts on anthocyanins (specifically the quinoidal base form) to generate phenolic acids and aldehydes [59].

Some studies suggest that anthocyanins may be converted into quinone-like intermediates during oral processing. These reactive compounds can bind to salivary proteins and mucins, reducing their lubricating capacity and promoting protein aggregation. Such interactions are believed to contribute to the perception of astringency frequently associated with anthocyanin-rich foods [63]. Catechol-O-methyltransferase (COMT) plays a role in modifying anthocyanins during the initial phases of digestion in the oral cavity. For example, COMT catalyze methylation at the 3′ position of cyanidin-3-glucoside to convert it to peonidin-3-glucoside [59]. Another enzyme, arylsulfatase, which is detected in human saliva, plays a role in anthocyanin metabolite recycling by removing sulfate groups previously introduced by sulfotransferases [59]. The absence of sulfotransferase expression in oral epithelial tissues suggests that sulfation–desulfation cycling in the oral cavity is primarily mediated by salivary and microbial enzymatic activity rather than host epithelial metabolism [59]. Extracts or juices obtained from berries may enhance the immediate release of anthocyanins, reduce their digestive stability, and eliminate the protective food matrix [53]. The anthocyanin metabolites available at the end of digestion ultimately determine their absorption, cellular uptake, and microbial metabolism. Those processes collectively contribute to their physiological effects. Therefore, matrix–compound interactions are important for the development of functional foods, and nutraceutical delivery systems should be designed to optimize anthocyanins’ bioavailability [63].

### 4.2. Anthocyanins’ Stability and Bioaccessibility in the Stomach

In their review, Gui et al. (2023) [62] highlighted that the stomach represents a favorable environment for anthocyanin stability and early absorption due to its acidic pH conditions (1.5–3 pH value), depending on the food matrices, which maintain anthocyanins predominantly in their structurally stable flavylium cation form [62]. Unlike many dietary polyphenols, anthocyanins can be absorbed directly from the stomach without prior hydrolysis, with approximately 20–25% of ingested anthocyanins undergoing rapid gastric absorption and appearing in plasma within 30 min after intake [62]. Typically, peak plasma concentrations in anthocyanins are reached within 0.5–2 h, indicating that gastric kinetics play a critical role in determining absorption efficiency. However, because of their hydrophilic nature and relatively high molecular weight (~500 g/mol), anthocyanins cannot efficiently cross the gastric epithelium via passive diffusion [62]. Instead, their absorption occurs primarily through carrier-mediated transport mechanisms involving specific membrane transporters. Among these, bilitranslocase plays a central role as an organic anion transporter expressed in gastric epithelial cells, facilitating the uptake of intact anthocyanin glycosides based on their structural affinity, particularly their glycosylation pattern [21]. In addition, glucose transporters, including GLUT1, GLUT3, and sodium-dependent glucose transporter (SGLT1), contribute to anthocyanin uptake, reflecting the structural similarity between anthocyanin glycosides and glucose-containing substrates. Other transport systems implicated in gastric anthocyanins transport include organic cation transporter 1 (OCT1), organic anion transporter 2 (OAT2), and sodium-coupled monocarboxylate transporters (SMCT1 and SMCT2), highlighting the involvement of multiple carrier-mediated pathways [65]. Anthocyanins’ absorption efficiency in the stomach is strongly influenced by molecular structure. Research suggests that glycosylation enhances recognition by transporters, whereas excessive glycosylation or structural modifications, such as glucuronidation or acylation, may reduce transport efficiency due to steric hindrance or reduced transporter affinity [66]. Among anthocyanidins, cyanidin exhibits greater stability and transport efficiency compared to more hydroxylated structures such as delphinidin and petunidin. This reflects the influence of hydroxylation on molecular stability and absorption. Also, anthocyanin transport is time-dependent and saturable, indicating competition for shared transport systems. This aspect is more obvious when anthocyanins are consumed as part of complex food matrices [67]. Studies using the MKN-28 cell (human gastric epithelial model) provide clear mechanistic evidence regarding the transporter-mediated absorption of anthocyanins at the gastric level [67]. Saturable kinetics, characterized by Michaelis–Menten behavior, indicate that anthocyanins interact with selective carrier systems that can become saturated at higher substrate concentrations, thereby limiting further increases in transport rate [67]. This mechanism fits the physicochemical properties of anthocyanins, which exist predominantly as hydrophilic and positively charged flavylium cations under acidic gastric conditions, making passive diffusion across lipid membranes unlikely [67]. The presence of glycosylated moieties, particularly glucose residues, plays a critical role in facilitating anthocyanin uptake. Gastric epithelial cells express glucose-related transporters such as GLUT1 and GLUT3, and their inhibition has been shown to significantly reduce anthocyanin transport. In addition, bilitranslocase, an organic anion transporter expressed in gastric tissue, has been proposed as an important carrier facilitating the uptake of intact anthocyanin glycosides [67]. Anthocyanins’ structural differences, like glycosylation pattern and substitution on the B-ring, further influence transporter affinity and absorption efficiency. Collectively, these findings confirm that anthocyanin absorption in the stomach occurs through a regulated, transporter-dependent process, highlighting the gastric epithelium as an active and selective site contributing to the early systemic availability of intact anthocyanins [67].

Unlike in the oral cavity, enzymatic degradation in the stomach is minimal (Table 3), and anthocyanins are largely absorbed as intact glycosides rather than being extensively metabolized. Overall, the stomach functions as an important absorption site. Here, anthocyanin stability is preserved and uptake is mediated primarily by specialized transport proteins rather than enzymatic transformation. These findings highlight gastric transport as a critical determinant of anthocyanin bioavailability and suggest that factors prolonging gastric residence time may enhance systemic exposure to intact anthocyanins [20].

### 4.3. Intestinal Fate and Absorption of Anthocyanins

**Figure 3:**
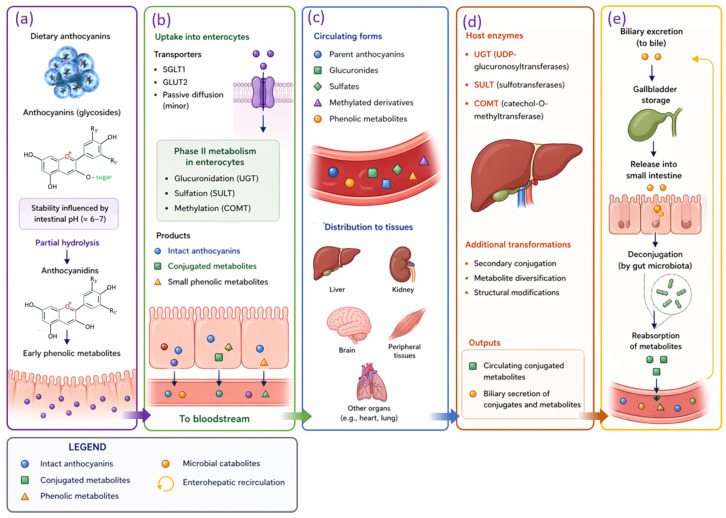
Diagram of the small intestine circulation and enterohepatic circuit of anthocyanins and their metabolites, with an emphasis on blueberry anthocyanins. Gastrointestinal release and luminal transformation (**a**). Intestinal absorption and enterocyte biotransformation (**b**). Systemic circulation and tissue distribution (**c**). Hepatic metabolism (phase II) (**d**). Enterohepatic recirculation (**e**).

Evidence indicates that anthocyanins can persist after passage through the stomach and undergo significant absorption in the small intestine through specific physiological mechanisms. The intestinal epithelium is coated by a protective mucus layer that is synthesized and secreted by specialized goblet cells [70,71]. This mucus consists predominantly of highly glycosylated mucin proteins, which assemble into a viscoelastic gel-like barrier. This mucin-rich layer serves as a primary defense mechanism by limiting direct contact between luminal microorganisms and epithelial cells. Moreover, studies in murine models have shown that nearly 80% of administered anthocyanins can be recovered from the small intestine within two hours following gastric delivery, probably due to a strong interaction with intestinal mucin [8]. This interaction potentially modulates rapid degradation and contributes to delayed metabolic processing of these intact compounds [72]. When present at high concentrations, anthocyanins may partially escape first-pass metabolism because of the saturation of metabolic pathways. In the small intestine enterocytes, intact anthocyanin glycosides can enter via the sodium-coupled glucose transporter (SGLT1) without deglycosylation. Intracellular hydrolysis is mediated by cytosolic β-glycosidase for some anthocyanins [55]. Alternatively, deglycosylation may occur at the apical membrane through lactase–phlorizin hydrolase (LPH), allowing absorption of the aglycone form. Both SGLT1 and LPH are located on the apical surface of enterocytes in the small intestine, facilitating efficient uptake of anthocyanins [8]. β-glucuronidase catalyzes the hydrolysis of anthocyanin–glucuronide conjugates [37]. Following intestinal absorption, anthocyanins and their aglycone forms are taken up by enterocytes, where they undergo extensive phase II metabolism to enhance their solubility, facilitate systemic transport, and promote elimination [8]. These biotransformation processes primarily involve conjugation reactions catalyzed by specific enzymes, including UDP-glucuronosyltransferases (UGTs), which mediate glucuronidation; sulfotransferases (SULTs), which catalyze sulfation; and catechol-O-methyltransferase (COMT), which is responsible for methylation (Table 3). Glucuronidation and sulfation increase the hydrophilicity of anthocyanins, thereby improving their transport in biological fluids and facilitating target organ activity, while methylation may enhance their lipophilicity and promote tissue distribution and retention [42]. In addition to enzymatic modification, membrane transporters play a critical role in the absorption and disposition of anthocyanins (Figure 3). The sodium-dependent glucose co-transporter 1 (SGLT1) contributes to the uptake of glycosylated anthocyanins across the intestinal brush border, whereas efflux transporters, including multidrug resistance-associated protein 2 (MRP2), P-glycoprotein (P-GP), and breast cancer resistance protein (BCRP), regulate the export of conjugated metabolites from enterocytes into the intestinal lumen or systemic circulation [73]. Collectively, these enzymatic and transporter-mediated processes determine the bioavailability, distribution, and biological activity of anthocyanins in the organism [62]. Studies on Ussing chamber systems demonstrated that anthocyanin absorption is highest in the jejunum, identifying the small intestine as a major site of uptake and enterohepatic recycling. Additionally, methylated and glucuronidated phase II conjugates are rapidly excreted into bile and may be deconjugated by host or microbial enzymes in the small intestine, enabling reabsorption and prolonging their systemic presence through enterohepatic circulation [74].

### 4.4. The Enterohepatic Circulation of Anthocyanins

Enterohepatic circulation plays a critical role in prolonging the biological presence, metabolic transformation, and bioavailability of anthocyanins and their derivatives [62]. In hepatocytes, anthocyanin metabolites undergo additional conjugation and are subsequently excreted into the bile via efflux transporters (Figure 3), allowing their re-entry into the small intestine, particularly the jejunum [62]. Regarding the subsequent metabolism of anthocyanidins, studies have shown that human liver microsomes can convert cyanidin into PCA, which is further processed into glucuronide conjugates [55]. In a similar manner, pelargonidin is metabolized to 4-hydroxybenzoic acid, followed by its transformation into two additional glucuronide conjugates through hepatic microsomal activity [55]. In the intestinal lumen, these metabolites may be deconjugated by intestinal or microbial enzymes, reabsorbed into enterocytes, or further degraded by the gut microbiota into low-molecular-weight phenolic compounds, including protocatechuic acid, ferulic acid, PGA, and hippuric acid [62].

This continuous cycle of conjugation, biliary excretion, microbial modification, and reabsorption may occur multiple times, generating mono-, di-, and polyconjugate derivatives and hybrid host–microbial metabolites. These processes significantly extend the residence time of anthocyanins and their metabolites in the organism, enhance their systemic availability, and contribute to their sustained biological activity, thereby supporting the concept that anthocyanins exhibit greater bioavailability and metabolic persistence than previously assumed [62].

### 4.5. The Modulation of Anthocyanins on the Colon Microbiome

Anthocyanins that escape absorption in the upper gastrointestinal tract reach the colon, where they interact with gut microbiota and undergo enzymatic degradation. Through this process, they are transformed into phenolic acids, such as protocatechuic acid, gallic acid, and vanillic acid, which can exert beneficial biological effects in different tissues of the body [51]. For instance, active phenolic compounds can travel through the blood–retina barrier, offering anti-inflammatory and antioxidant activities and leading to potential modulation [75].

**Figure 4:**
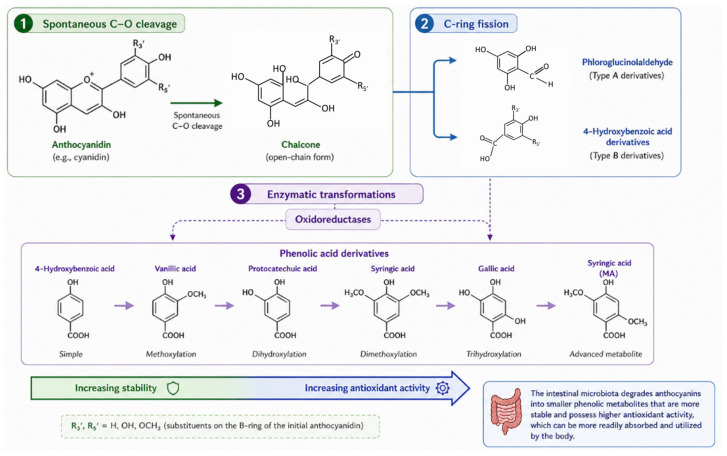
Proposed biotransformation pathways of anthocyanins in the human gut microbiota.

In particular situations, dietary anthocyanins glycosylated with infrequent monosaccharides and their derivatives, like acylated forms, cannot be hydrolyzed by β-glycosidase produced by the cells of the OGIB. Those compounds are more exposed to β-glycosidases produced by colon bacteria [8]. Abundant gut microbiota produces enzymes like β-glucosidase, α-rhamnosidase, and esterases to break down complex anthocyanins, leading to deglycosylation and B-ring fission (Figure 4) [76].

Recently, some in vitro studies using a human feces-inoculated pH-controlled colon model have confirmed that anthocyanins and anthocyanidins are subjected to intensive degradation through a hybrid process [76]. This degradation involves a combination of two distinct processes: (1) spontaneous degradation due to the instability of anthocyanins at near-neutral pH (6.6–7.0) and anaerobic conditions characteristic of the human colon; (2) microbiota-dependent degradation through metabolic activity and enzyme production by gut bacteria [76]. More specifically, the cyanidin and cyanidin-3-O-glucoside colon metabolites are often obtained by the hybrid degradation process with PGA, PCA, vanillic acid, ferulic acid, caffeic acid, 3,4-dihydroxiphenylacetic acid, and 4-hydroxiphenylacetic acid [76]. In this condition, the colon cells can be protected against oxidative stress by those anthocyanins and their metabolites, with other antioxidants being almost absent in the colon [8]. The proposed pattern regards anthocyanin biotransformation in human gut microbiota to PGA and 4-hydroxi-benzoic acid derivatives (Figure 4). The increase in antioxidant activity occurs with the increase in the number of –OH groups; the stability of the structure increases with the increase in the number of CH_3_– groups (Figure 4).

Other research studies regarding administration of labeled ^13^C cyanidin 3-glucoside to humans have revealed that around 30% of the total ingested concentration reached the colon lumen, with the rest being absorbed in the stomach and upper part of the small intestine [55]. Also, 41% of delphinidin 3-glucoside was found in the colon [55].

**Figure 5:**
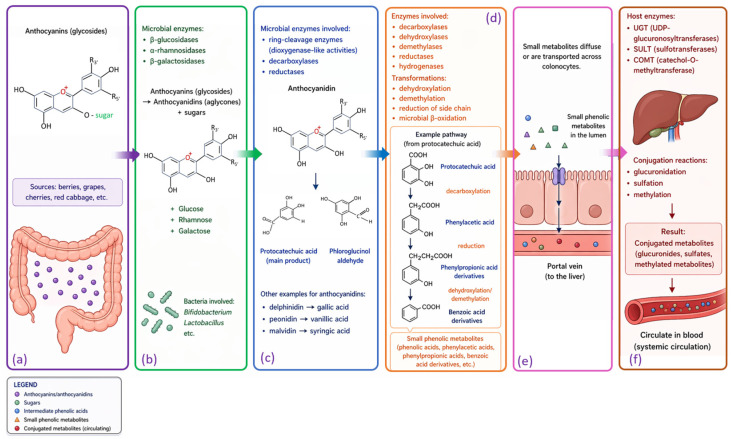
Schematic representation of anthocyanidin degradation in the colon and the metabolic fate of derived metabolites, with an emphasis on blueberry anthocyanins. Unabsorbed anthocyanins reach the colon (**a**). Anthocyanin glycosylation by gut microbiota (**b**). Ring fission of flavylium nucleus by gut microbiota (**c**). Secondary microbial metabolism (**d**). Colonic absorption of anthocyanin metabolites (**e**). Hepatic metabolism (phase II) (**f**).

Microorganisms can transform into metabolites from the intact anthocyanins that reach the colon lumen (Figure 5). A study on ileal fluid showed that malvidin glycosides are found in the highest concentration, followed by petunidin glycosides and delphinidin glycosides; peonidin glycosides and cyanidin glycosides were detected in the lowest concentration [77]. In addition, both anthocyanins and their metabolites contribute to gut health by modulating intestinal barrier function and promoting the growth of beneficial microbial species. These changes enhance resistance to pathogenic microorganisms, improve nutrient metabolism, and support the overall immune response [51].

The human colonic microbiota is a highly complex and dynamic ecosystem primarily composed of four major phyla—*Actinobacteria* (now often *Actinomycetota*), *Bacillota* (formerly *Firmicutes*), *Bacteroidetes* (now often *Bacteroidota*), and *Proteobacteria* (now *Pseudomonadota*)—with the first two together accounting for approximately 90% of the total microbial population [78]. Although present in relatively lower abundance, beneficial bacterial genera such as *Bifidobacterium* spp. and *Lactobacillus* spp. play a crucial role in maintaining gut health [78]. The composition of the gut microbiome is dynamic and evolves in response to both internal and external factors. Maintaining a balanced and diverse microbial community is essential for normal gastrointestinal function, including the production of AC metabolites and short-chain fatty acids, regulation of lipid metabolism, support of immune defense mechanisms, and modulation of the gut–eye axis [5,51]. For example, an alteration in the *Bacillota*-to-*Bacteroidetes* ratio has been associated with AMD patients [6], while a reduction in *Lactobacillus* species has been observed in favor of species that promote pro-inflammatory effects [79]. Also, anthocyanins positively influence gut microbiota, indirectly contributing to eye health [33].

## 5. Mechanisms of Ocular Protection Potentially Modulated by Anthocyanins and Their Metabolites

### 5.1. Direct Ocular Effects of Anthocyanins and Their Metabolites

**Figure 6:**
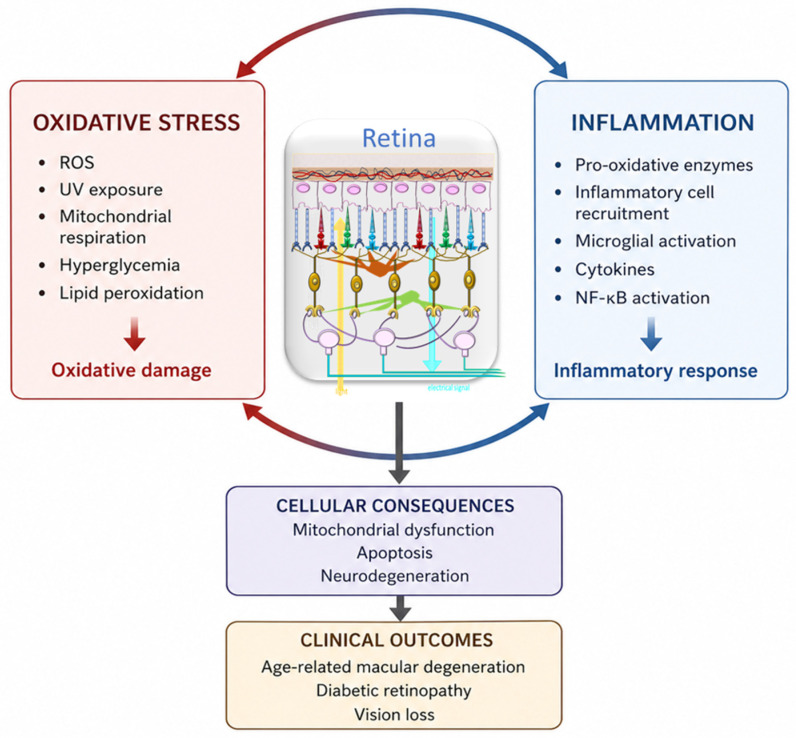
Major consequences of the vicious cycle between oxidative stress and inflammation. Oxidative stress and inflammation mutually reinforce each other in the retina, contributing to cellular damage, apoptosis, neurodegeneration, and retinal disorders.

The oral–gastrointestinal passage presented in the third part of this review identified multiple degradation products of the intact anthocyanidin in humans. Primarily, compounds like PGA and 4-hydroxibenzoic acid derivatives represent the major circulating phenolic metabolites. Secondly, the gut microbiota and host metabolism cooperatively transform anthocyanins into metabolites that are absorbed into the bloodstream and undergo enterohepatic circulation, thereby enhancing their systemic persistence and bioavailability [20]. Beyond their systemic metabolic effects, the ability of glycosylated anthocyanins to modulate glucose digestion and intestinal absorption may be indirectly relevant to eye health. By inhibiting α-amylase and α-glucosidase activity and competitively reducing glucose uptake through SGLT1 and GLUT2 transporters in the small intestine, anthocyanin glycosides attenuate postprandial hyperglycemia [80]. This regulatory effect on blood glucose levels is particularly important in the context of ocular complications associated with metabolic disorders, such as diabetic retinopathy, where chronic hyperglycemia promotes oxidative stress, microvascular damage, and inflammation within retinal tissues (Figure 6).

Therefore, although only a limited fraction of anthocyanins is absorbed intact, their capacity to improve glycemic control at the intestinal level may indirectly contribute to retinal protection [31]. In addition to their direct antioxidant and anti-inflammatory effects in ocular tissues, the modulation of systemic glucose homeostasis represents an important complementary mechanism through which anthocyanins may support long-term eye health [80]. Recent advances in microbiome research have highlighted the existence of a gut–eye axis, providing a novel framework for understanding the link between intestinal homeostasis and ocular health. Gut dysbiosis has been associated with the development and progression of retinal disorders through multiple mechanisms, including altered production of microbial metabolites, immune dysregulation, and impaired intestinal barrier integrity [7]. In particular, reduced levels of short-chain fatty acids (SCFAs) and increased systemic exposure to pro-inflammatory molecules contribute to chronic low-grade inflammation, which may negatively affect retinal pigment epithelium function and exacerbate oxidative stress [79]. Experimental studies further support a causal relationship, demonstrating that alterations in gut microbiota can modulate retinal inflammation. In this context, dietary compounds such as anthocyanins may play a dual role, not only through their direct antioxidant effects in ocular tissues but also indirectly by modulating gut microbiota composition and metabolic activity, thereby contributing to the maintenance of the gut–eye axis [5].

Together with this biological background, modern lifestyle factors have emerged as significant contributors to ocular health outcomes. The rapid expansion of digital device use, including smartphones and tablets, has been accompanied by a notable increase in visual impairment, particularly in the context of prolonged screen exposure [17]. In this regard, anthocyanins derived from blueberries have attracted considerable attention due to their ability to enhance visual sensitivity and support ocular health. Some of the experimental and clinical evidence supporting the ocular benefits of blueberry anthocyanins is presented in Table 4.

The available evidence regarding the ocular effects of blueberry anthocyanins originates from different levels of investigation, including in vitro experiments, animal studies, observational studies, and a limited number of human intervention trials. These study designs provide complementary information but differ substantially in their ability to establish physiological relevance and clinical efficacy. Therefore, the findings should be interpreted according to the level of evidence available.

Growing evidence from in vitro, in vivo, and population-based studies indicates that anthocyanins exert protective effects on retinal structures through multiple mechanisms [18,33,81]. At the cellular level, studies on retinal pigment epithelium (RPE) and retina endothelial cells have shown that anthocyanins reduce oxidative stress by lowering reactive oxygen species (ROS) levels, while also suppressing pro-inflammatory and angiogenic mediators such as vascular endothelial growth factor (VEGF), ICAM-1, and NF-κB. These effects are often accompanied by the activation of endogenous antioxidant defense systems, including increased activity of enzymes such as superoxide dismutase (SOD) and catalase (CAT), as well as the modulation of key signaling pathways such as Nrf2/HO-1 [15].

Studies indicate that malvidin-3-glucoside and malvidin-3-galactoside, which are anthocyanins found in blueberries, act as anti-angiogenic agents by suppressing VEGF levels [81]. Results from a cross-sectional study indicate that higher intake of anthocyanins is associated with a significant reduction in the incidence of myopia among teenagers (ages 12–18), an effect observed for both total intake and the specific subtypes cyanidin, petunidin, and delphinidin. Probable biological mechanisms include antioxidant and anti-inflammatory properties, protection of retinal cells against oxidative stress and apoptosis, stimulation of rhodopsin regeneration, and regulation of ocular growth by influencing signaling pathways of the retinal pigment epithelium [33]. In addition, anthocyanins may improve retinal perfusion and positively influence the gut microbiota, indirectly contributing to eye health. Protective effects are modulated by factors such as sex, physical activity, body weight, and smoking, highlighting possible synergistic interactions between diet and lifestyle. These results suggest that including anthocyanin-rich foods, such as berries, could represent a non-invasive and cost-effective preventive strategy for reducing the risk of myopia in adolescents. However, the study’s limitations, including its cross-sectional design, intake estimation based on dietary data, and potential uncontrolled confounding factors, indicate the need for prospective and interventional studies to confirm the causal relationship between anthocyanin consumption and myopia prevention [33].

### 5.2. Indirect Ocular Effects Mediated Through the Gut–Eye Axis

Among anthocyanin-rich foods, blueberries are of particular interest for ocular health because they contain a broad spectrum of anthocyanin glycosides, including malvidin-, delphinidin-, petunidin-, peonidin-, and cyanidin-derived compounds [49]. Several of these molecules and their metabolites have been associated with retinal antioxidant protection, vascular regulation, and modulation of inflammatory pathways [50]. Furthermore, blueberries have been more extensively investigated in ophthalmological studies than most other dietary anthocyanin sources, making them a relevant model for understanding the interactions between anthocyanin metabolism, gut microbiota activity, and retinal function [51].

The retina is a complex, multilayered, light-sensitive tissue and a functional extension of the central nervous system [83]. The choroid, a highly vascularized layer, supplies oxygen and nutrients to the outer retina; dysfunction of this vascular network, including increased permeability and neovascularization, contributes to retinal disorders such as AMD and diabetic retinopathy [5]. In vitro, blueberry-derived anthocyanins have been shown to exert vasoprotective effects by improving endothelial function, reducing oxidative stress, and modulating angiogenic signaling pathways (e.g., VEGF), thereby contributing to the maintenance of choroidal vascular integrity [81].

The RPE, a monolayer of highly specialized cells, plays a critical role in photoreceptor support, including nutrient transport, phagocytosis of photoreceptor outer segments, and clearance of metabolic waste [83]. Dysfunction of the RPE, particularly impaired degradation of photoreceptor debris and accumulation of lipofuscin, is a hallmark of degenerative retinal diseases such as AMD and Stargardt disease [84]. In in vitro studies, anthocyanins and their metabolites have been reported to protect RPE cells by attenuating oxidative stress, inhibiting inflammatory signaling (e.g., NF-κB), and enhancing cellular antioxidant defenses, thereby supporting RPE homeostasis and function [85].

**Figure 7:**
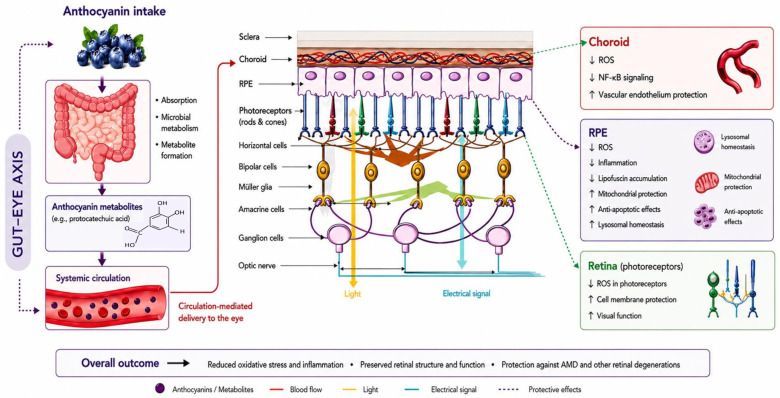
Schematic representation of the gut–eye axis mediating anthocyanin-driven ocular protection. (↑ enhance; ↓ decrease).

Photoreceptors (rods and cones) are responsible for phototransduction, converting light into electrical signals that are transmitted via retinal neurons to the brain (Figure 7). These cells are particularly vulnerable to oxidative damage due to their high metabolic activity and exposure to light [86]. In in vitro studies, blueberry anthocyanins have been shown to reduce reactive oxygen species (ROS) levels and improve photoreceptor cell viability, contributing to the preservation of retinal function [87]. Evidence from observational studies suggests that anthocyanins may enhance rhodopsin regeneration, a key process in the visual cycle, thereby supporting visual performance, particularly under low-light conditions [17].

At the molecular level, visual pigments such as rhodopsin are formed by the binding of opsins to the chromophore 11-cis-retinal [88]. Upon light exposure, 11-cis-retinal is converted to all-trans-retinal, initiating the phototransduction cascade. Efficient recycling of all-trans-retinal back to 11-cis-retinal in the RPE is essential for sustained visual function. Anthocyanins have been suggested to facilitate this visual cycle, potentially by stabilizing photoreceptor membranes and supporting enzymatic processes involved in chromophore regeneration [88].

The bioactivity of blueberry anthocyanins at the retinal level is strongly influenced by the selective permeability of the blood–retina barrier (BRB), a highly specialized structure analogous to the blood–brain barrier (3B) [81]. The BRB consists of two main components: the inner barrier, formed by retinal capillary endothelial cells, and the outer barrier, represented by the RPE [89]. While this barrier plays a critical role in maintaining retinal homeostasis by protecting neural tissue from circulating toxins and systemic fluctuations, it also limits the penetration of many bioactive dietary compounds, including anthocyanins [81].

Due to their relatively large molecular size and chemical instability, anthocyanins cross the BRB only to a limited extent [42]. However, various conjugated and degraded metabolites exhibit improved tissue accessibility compared to the intact anthocyanins, facilitating their partial transport into ocular tissues [9]. Accumulation appears to be more pronounced in highly vascularized structures such as the choroid, as well as in the RPE, whereas only low concentrations are detected in the neural retina [17]. Nevertheless, even these low levels are considered biologically relevant [90].

Importantly, the effects of anthocyanins on the retina are predominantly functional rather than structural, enhancing dark adaptation, reducing eye fatigue, and protecting against light-induced damage. These compounds do not accumulate permanently in ocular tissues; instead, they exert transient and dynamic biological effects [17,85]. Their biological activity is mediated through multiple mechanisms, including potent antioxidant and anti-inflammatory effects, even at low concentrations. In addition, anthocyanins may indirectly support retinal function by improving ocular microcirculation, thereby enhancing oxygen and nutrient delivery to retinal cells [90].

At the cellular level, anthocyanins and their metabolites play a particularly important role in the RPE, where they modulate oxidative stress, reduce local inflammation, and support key processes such as photopigment recycling [77]. Furthermore, they are known to influence intracellular signaling pathways, including the activation of nuclear factor erythroid 2-related factor 2 (Nrf2), which enhances antioxidant defense systems, and the inhibition of nuclear factor kappa B (NF-κB), a central regulator of inflammation [91]. Through these combined mechanisms, blueberry anthocyanins contribute to the maintenance of retinal homeostasis and may exert protective effects against the development and progression of retinal diseases [7,79,87].

#### Molecular Mediators of Gut–Eye Communication

The gut–eye axis is mediated by a complex network of microbial metabolites, immune mediators, and metabolic signaling molecules that link intestinal homeostasis with retinal function. Among the most extensively studied mediators are short-chain fatty acids (SCFAs), bile acid derivatives, and inflammatory cytokines [79]. SCFAs, including acetate, propionate, and butyrate, are produced through microbial fermentation of dietary fibers and polyphenol-derived substrates in the colon [79]. These metabolites exert systemic anti-inflammatory effects by regulating regulatory T-cell differentiation, suppressing pro-inflammatory cytokine production, and enhancing intestinal barrier integrity. Through these mechanisms, SCFAs may reduce systemic inflammation and indirectly protect retinal tissues from inflammatory damage [79].

Bile acids represent another important class of signaling molecules within the gut–eye axis. Gut microbiota convert primary bile acids into secondary bile acids, which act as ligands for nuclear receptors such as farnesoid X receptor (FXR) and Takeda G-protein-coupled receptor 5 (TGR5) [92]. Activation of these pathways influences glucose metabolism, lipid homeostasis, oxidative stress responses, and inflammatory signaling, all of which have been implicated in retinal disorders including diabetic retinopathy and age-related macular degeneration [92].

Inflammatory mediators constitute a third major communication pathway. Gut dysbiosis may increase intestinal permeability, facilitating the translocation of microbial products such as lipopolysaccharides (LPS) into systemic circulation [10,11,12]. This process can stimulate the production of pro-inflammatory cytokines, including tumor necrosis factor-alpha (TNF-α), interleukin-1β (IL-1β), and interleukin-6 (IL-6), which may contribute to retinal inflammation, microglial activation, vascular dysfunction, and breakdown of the blood–retina barrier [70,89].

By modulating gut microbial composition and metabolic activity, anthocyanins may influence the production of these signaling molecules and thereby indirectly contribute to the maintenance of retinal homeostasis through gut–eye communication [5,6].

## 6. Current Gaps, Limitations, and Future Perspectives

While the intersection of dietary anthocyanins, the colonic microbiome, and ocular function present a compelling therapeutic framework, a critical evaluation of the literature reveals distinct methodological limitations that future research must address before clinical translation is viable (Table 5).

## 7. Conclusions

Ocular disorders such as age-related macular degeneration, diabetic retinopathy, or progressive myopia represent major causes of visual impairment worldwide, highlighting the need for complementary multi-targeted strategies to support long-term ocular health. In this context, dietary anthocyanins have emerged as promising nutritional adjuvants due to their antioxidant, anti-inflammatory, vasoregulatory, and neuroprotective properties, with blueberry profiles serving as an exceptionally rich and well-documented model matrix.

The evidence reviewed in this paper suggests that the clinical and biological efficacy of the anthocyanins depends not only on the parent compounds but also on the metabolites generated through gastrointestinal digestion, host metabolism, and gut microbial biotransformation. Although the systemic bioavailability of intact anthocyanins is relatively low, their extensive breakdown and remodeling by both host enzymes and the colonic microbiota generate numerous active phenolic metabolites capable of exerting both systemic and local ocular protective effects.

The gut–eye axis provides an emerging mechanistic framework linking anthocyanin metabolism, gut microbiota modulation, and retinal homeostasis. Through the regulation of microbial composition, microbial metabolites, inflammatory signaling, and metabolic pathways, anthocyanins and their derived metabolites may influence ocular health both indirectly through systemic mechanisms and directly through protective actions on retinal cells and vasculature.

However, much of the mechanistic evidence currently available remains limited to specific experimental models, notably those utilizing blueberry extracts. Therefore, dietary anthocyanins should be considered adjunctive nutritional modulators rather than alternatives to established, evidence-based ophthalmological therapies. Future well-designed, long-term clinical trials and translational studies are strictly required to clarify their therapeutic efficacy, pinpoint the most relevant circulating bioactive metabolites, and further elucidate the precise molecular mechanisms underlying gut–eye communication.

## Figures and Tables

**Table 1:** Current therapeutic modalities for retinal disorders: mechanisms, limitations, and clinical challenges.

Therapeutic Modality	Primary Target/Mechanism	Major Limitations	Clinical Challenges	Reference
**Anti-VEGF Therapy**	Neutralization of extracellular VEGF isoforms to suppress angiogenesis and reduce vessel permeability	Short half-life requiring lifelong intravitreal injections; risk of geographic atrophy with chronic suppression; 30–40% of patients show sub-optimal response	Endophthalmitis risk, high financial burden, and patient non-compliance due to injection anxiety	[3]
**Intravitreal Corticosteroids**	Broad suppression of inflammatory cytokines, leukostasis, and VEGF synthesis	Non-selective action altering fluid dynamics and trabecular meshwork extracellular matrix	Accelerated cataractogenesis; secondary ocular hypertension and open-angle glaucoma	[39]
**Laser Photocoagulation**	Ablation of hypoxic peripheral retina to lower total ocular oxygen demand; direct sealing of leaking microaneurysms	Permanent, irreversible destruction of healthy neural retinal tissue	Reduction in peripheral visual field; permanent loss of night vision (nyctalopia); transient macular edema	[40]

**Table 2:** Enzymes involved in the biotransformation of anthocyanins in the oral cavity and their associated reaction products and biological consequences, with an emphasis on blueberry anthocyanins.

	Enzyme	Reaction Type	Main Reaction Products	Biological Consequence	References
**Anthocyanin oral cavity enzyme transformation**	β-glucosidase	deglycosylation	unstable anthocyanidins	↑ lipophilicity ↑ absorption through the oral mucosa ↑ chalcone glucosides (via C-ring opening) → PCA & PGA ↑ flavor perception and sensitivity	[59,62,64]
	lactase phlorizin hydrolase	deglycosylation	anthocyanidins	↑ metabolic reaction	[59,62]
	quinoid anhydrolase	cleavage of the heterocyclic C-ring	PGA and PCA	↑ smaller molecules to be easily absorbed ↑ antioxidant or anti-inflammatory protection to the oral mucosa ↑ sensation of astringency	[59]
	β-glucuronidase	deglycosolation	anthocyanidin glucuronic acid	↑ absorption through the oral mucosa ↑ instability	[59,62]
	arylsulfatase	hydrolysis of aryl sulfate esters	anthocyanidin and sulfate ions (SO_4_^2−^)	↑ structural modification of polyphenols upon consumption	[59,62]
	catechol-O-methyltransferase	methylation	methylated anthocyanin conjugates	↑ anthocyanins interconversion ↑ bioavailability/bioactivity	[59,62]
	UDP-glucose-dehydrogenase	NAD^+^ dependent oxidation	glucuronic acid	↑ glucuronic acid for UGTs	[59,62]
	UDP-glucuronosyl transferase (UGTs)	glucuronidation	anthocyanin glucuronide conjugates	↑ solubility ↑ retention of anthocyanin glucuronides and detoxification process in the oral tissues	[59,62]

↑ indicates an increase in the parameter value; phloroglucinol aldehyde (PGA); protocatechuic acid (PCA); UDP-glucuronosyl transferase (UGTs).

**Table 3:** Enzymes involved in biotransformation of anthocyanins in the gastrointestinal tract, with an emphasis on blueberry anthocyanins.

	Enzyme	Reaction Type	Main Reaction Products	Biological Consequence	References
**Anthocyanin stomach enzyme transformation**	UDPs-glucuronosyltransferases,	glucuronidation	anthocyanin glucuronide conjugates	↑ anthocyanin hydrophilicity ↑ bioavailability and systemic bioactivity ↑ excretion	[36,67]
	SULTs	sulfation	anthocyanin sulfated conjugates	↑ distribution across cellular barriers protect scavenging capacity until ACs get to the inflammation site	[67,68]
	catechol O-methyl transferase	methylation	methylated anthocyanin conjugates	↓ AC degradation in the stomach ↑ interaction with transporters like bilitranslocase	[67,68]
**Anthocyanin small intestine enzyme transformation**	β-glucosidase	deglycosylation	anthocyanidins	↑ hydrophobic character ↑ absorption ↑ metabolic conversion	[8]
	lactase phlorizin hydrolase	deglycosylation	anthocyanidins	↑ hydrolysis on apical side of the enterocyte	[8]
	catechol-O-methyltransferase	methylation	methylated anthocyanin conjugates	↑ absorption from enterocyte into systemic circulation	[62]
	β-glucuronidase	deglucuronidation	anthocyanidins and β-D-glucuronic acid	↑antioxidant and anti-inflammatory activity ↑ intestinal barrier function ↓ gut oxidative stress	[51,69]
	SULTs	sulfation	sulfated anthocyanin conjugates	↑ solubility and excretion ↑ detoxification	[51]

↑ indicates an increase in the parameter value; ↓ indicates a decrease in the parameter value; sulfotransferases (SULTs).

**Table 4:** Summary of experimental and clinical evidence supporting the ocular benefits of blueberry anthocyanins.

Evidence Level	Experimental Model/Population	Anthocyanin Source	Main Findings Relevant to Ocular Health	References
**In vitro studies**	Human retinal capillary endothelial cells exposed to high glucose	Blueberry anthocyanin extract, 10 μg/mL BAE	↑ cell viability ┴ ↑ ROS ┴ ↑ Akt expression ↓ VEGF	[81]
		malvidin (Mv)	↑cell viability ┴ ↑ ROS ┴ HG-Nox4 NF-κB	
		malvidin-3-glucoside (Mv-3-glc)	↑ cell viability ┴ ↑ ROS ↓ VEGF [NO]: Δ ┴ **HG-ICAM-1** ┴ ↑ Akt expression	
		malvidin-3-galactoside (Mv-3-gal)	↑ cell viability ┴ ↑ ROS ┴ ↑ Akt expression	
**Observational human studies**	Middle-aged and older women	Blueberry intake (≥1 serving/wk)	28% reduction in total AMD	[18]
**Observational human studies**	Adolescents (12–18 years), NHANES dataset	Dietary anthocyanins, especially cyanidin, petunidin, and delphinidin	↓ incidence of myopia ↑ relaxing effect on ciliary muscle	[33]
**Human intervention studies**	Adults (20–59 years) with visual display terminal (VDT) fatigue	Active capsule (36 mg of anthocyanin, 3 mg of astaxanthin, and 5 mg of lutein)	↑ regeneration of rhodopsin ↓ production of ROS ↓ asthenopia ↓ age-related macular degeneration ↑ macular antioxidant capacity ↑ visual acuity	[82]

BAE—blueberry anthocyanin extract; AMD—age-related macular degeneration; HG—high glucose; Nox4—NADPH oxidase 4; VEGF—vascular endothelial cell growth factor; NO—nitric oxide; ICAM-1—intercellular adhesion molecule-1; NF-κB—nuclear factor-kappa B; VDT—visual display terminals; ↑ enhance; ↓ decrease; ┴ inhibit; Δ change.

**Table 5:** Critical evaluation of current methodological gaps and corresponding future solutions for the gut–eye axis.

Experimental Context	Current Methodological Gaps and Limitations	Proposed Future Solutions and Directions	References
**In Vitro Studies and Pharmacokinetics**	Cell models often use parent compounds at supra-physiological doses (10–50 microg/mL), omitting gut microbial biotransformation. True tissue quantification across the blood–retina barrier (BRB) in humans is entirely missing.	Shift research toward testing realistic physiological concentrations of metabolite mixtures (e.g., PCA and PGA) rather than native anthocyanins. Emphasize advanced pharmacokinetic modeling.	[85,93]
**Gut–Eye Framework**	Existing evidence relies heavily on cross-sectional correlations between microbial dysbiosis and retinal diseases (like AMD), failing to definitively establish direct causality.	Utilize mechanistic fecal microbiota transplantation (FMT) and germ-free animal models to isolate specific immune and chemical signaling pathways driving the axis.	[93,94]
**Inter-Individual Variability**	Biological heterogeneity in human gut bacterial enzymes leads to distinct “metabotypes,” meaning individuals can be “strong” or “poor” responders to blueberry intake.	Transition to precision nutrition using personal microbiome profiling. Explore the direct clinical administration of stabilized, pure downstream postbiotic metabolites to ensure reproducible results.	[95,96,97]
**Delivery Systems and Bioavailability**	Native anthocyanins suffer from high chemical instability and rapid degradation in the upper gastrointestinal tract, leading to exceptionally poor systemic bioavailability.	Prioritize the development of advanced micro/nano-encapsulation, nano-emulsions, and liposomal delivery matrices designed to shield compounds from gastric pH.	[96]
**Clinical Translation and Regulation**	Current human trials are limited, often focusing on small cohorts and subjective metrics like visual fatigue rather than hard clinical disease endpoints.	Execute robust, large-scale, randomized double-blind placebo-controlled human trials to establish standardized therapeutic dosages for international guidelines (FAO/WHO).	[65,98]

## Data Availability

No new data were created or analyzed in this study. Data sharing is not applicable to this article.

## Abbreviations

The following abbreviations are used in this manuscript:

AMD	Age-related macular degeneration
PCA	Protocatechuic acid
PGA	Phloroglucinol aldehyde
LPH	Lactase phlorizin hydrolase
UGTs	UDP-glucuronosyl transferase
COMT	Catechol-O-methyltransferase
GLUT1	Glucose transporter Isoform 1
SULTs	Sulfotransferases
EHC	Enterohepatic circulation
RPE	Retinal pigment epithelium
ROS	Reactive oxygen species
VEGF	vascular endothelial growth factor
SCFAs	Short-chain fatty acids
NF-κB	Nuclear factor-kappa B
VDT	Visual display terminals
Nrf2	Nuclear factor erythroid 2-related factor 2
